# Nucleolar targeting of lyssavirus P-protein is isoform- and phylogroup-specific

**DOI:** 10.1099/jgv.0.002214

**Published:** 2026-01-16

**Authors:** Gregory W. Moseley, Yilin Zhang, Cassandra T. David, Stephen M. Rawlinson

**Affiliations:** 1 Department of Microbiology, Biomedicine Discovery Institute, Monash University, Clayton, VIC, 3800, Australia; 2 Department of Biochemistry and Molecular Biology, Bio21 Molecular Science and Biotechnology Institute, The University of Melbourne, Melbourne, 3052, Australia

**Keywords:** nuclear trafficking, nucleolus, phosphoprotein, rabies, RNA virus

## Abstract

The nucleolus is a multifunctional hub and a common target of viral proteins, yet its role in infections by cytoplasmically replicating RNA viruses remains poorly defined. In rabies virus (RABV), the phosphoprotein (P-protein) isoform P3 localizes to nucleoli and inhibits rRNA biogenesis, whereas P1 lacks nucleolar targeting, even when forced into the nucleus. Here, we show that nucleolar targeting is an isoform- and phylogroup-specific property of lyssavirus P-proteins. Isoforms P3–P5 accumulate in nucleoli, whereas P1 and P2 are excluded. Comparative analyses revealed that P3 nucleolar targeting is conserved in phylogroup I but absent in phylogroup II lyssaviruses. Co-immunoprecipitation assays identified conserved interactions with nucleolin and nucleophosmin (NPM1) but divergent binding to Treacle and nucleolar and coiled-body phosphoprotein 1 (NOLC1). These findings define nucleolar targeting as a gain-of-function of truncated P isoforms, demonstrate its conservation across phylogroup I lyssaviruses and suggest broader engagement with membraneless compartments, highlighting potential therapeutic vulnerabilities.

The nucleolus, classically known for its role in ribosome biogenesis, is now understood to be a multifunctional hub involved in diverse processes such as cell cycle regulation, DNA-damage sensing and repair, telomere maintenance, gene expression and stress responses [1,2]. Importantly, nucleolar targeting is not virus-specific: proteins from DNA viruses, RNA viruses and retroviruses have all been shown to accumulate in this compartment [3,5]. It has been suggested that, for RNA viruses with compact genomes, nucleolar targeting may provide an efficient way to access multiple cellular pathways through a single protein–host interaction [3,4]. Despite this common phenomenon, the significance of viral protein localization to nucleoli remains poorly understood, particularly for cytoplasmically replicating RNA viruses, which must divert proteins away from their primary sites of replication in order to engage the nucleolus.

For rabies virus (RABV), the phosphoprotein (P-protein) is a multifunctional protein expressed as five isoforms (P1–P5) (Fig. 1a) generated by leaky ribosomal scanning [6,8]. The essential isoform P1 serves as a cofactor for the viral polymerase L-protein [9] and is predominantly cytoplasmic, whereas smaller isoforms differ in trafficking due to distinct nuclear localization (NLS) and nuclear export signals (NESs) [10,12] and are implicated in accessory functions such as immune evasion [13]. Previous studies have shown that the P3 protein of the laboratory-adapted strains Challenge Virus Standard 11 (CVS) and Nishigahara (Nish) localizes to the nucleolus [14,15]. In CVS, P3 binds the nucleolar proteins nucleolin (NCL) and Treacle [14,16] and inhibits rRNA biogenesis in both transfected and RABV-infected cells; this function is abolished in a nucleolar-impaired P3 mutant (K214A/R260A, referred to as KRm; Fig. 1a) [16]. Knockdown of NCL or Treacle significantly reduces virus production, highlighting functional importance [14,16]. Our recent work further established that P1 does not target nucleoli or other membraneless organelles formed by liquid–liquid phase separation (LLPS), even when forced into the nucleus, despite containing the entire P3 sequence [17]. In contrast, P3 associates with multiple LLPS-driven bodies and interacts with additional nucleolar proteins, including nucleophosmin (NPM1) and nucleolar and coiled-body phosphoprotein 1 (NOLC1). Importantly, both P1 and P3 undergo LLPS *in vitro*, but only P3 binds RNA, suggesting that RNA interaction underlies its selective recruitment to specific LLPS compartments [17]. Together, these findings reveal isoform-specific nucleolar functions and implicate RNA binding as a critical determinant of P3-driven targeting of host condensates.

**Fig. 1.**
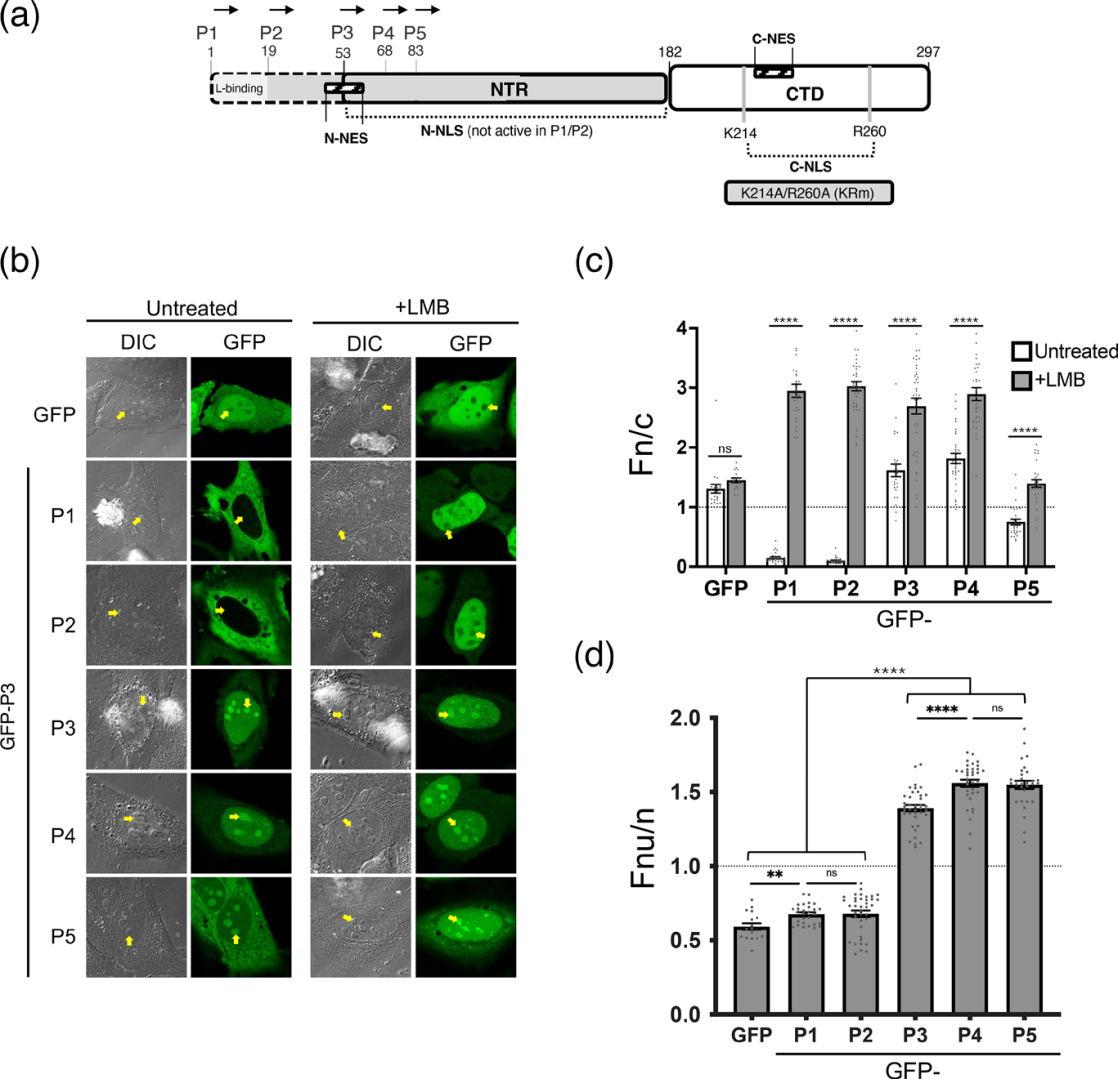
P-protein isoforms P3–P5 accumulate in nucleoli, whereas P1 and P2 are excluded. (**a**) Schematic of RABV P-protein isoforms showing start codons for P1, P2, P3, P4 and P5. The N-terminal region (NTR) and C-terminal domain (CTD) are indicated, together with known nuclear trafficking signals. Key residues K214 and R260 of the C-NLS, mutated to Ala (KRm), are highlighted and were previously shown to inhibit nucleolar targeting [16]. Residue numbering refers to full-length P1. (**b**) Confocal laser scanning microscopy (CLSM) images of live HeLa cells transfected with GFP-tagged P-protein isoforms using Lipofectamine 3000 (Thermo Fisher) and imaged 24 h post-transfection on a Leica SP5 confocal microscope (60× oil objective) [16,18]. Cells were treated with or without 5 µM leptomycin B (LMB) for 4 h. Panels show Differential Interference Contrast (DIC, left) and GFP fluorescence (right). Yellow arrows indicate nucleoli. (**c, d**) Quantitative image analysis of data from panels as in b. (**c**) Nuclear-to-cytoplasmic fluorescence ratios (Fn/c) were determined from ≥23 cells per condition. (**d**) Nucleolar-to-nuclear fluorescence ratios (Fnu/n) were determined following LMB treatment from ≥28 cells, except GFP alone (*n*=17). Data from c and d represent mean±sem from one representative assay and are consistent with three independent experiments. Statistical significance was determined using Student’s t-test; *P<0.05, **P<0.01, ***P<0.001, ****P<0.0001. NTR, N-terminal region; DIC, Differential Interference Contrast.

However, several key questions remain. It remains unclear whether other P isoforms beyond P3 have the capacity to localize to nucleoli or whether nucleolar targeting is conserved among lyssaviruses more broadly. Moreover, the conservation of interactions with nucleolar proteins such as NCL, NPM1, NOLC1 and Treacle across different viral lineages has not been addressed. To resolve these questions, we first examined the subcellular localization of GFP-tagged P1–P5 from the Nish strain in HeLa cells and imaged by confocal laser scanning microscopy (CLSM) (noting that P3 nucleolar localization has previously been demonstrated using untagged and small epitope-tagged constructs [17], indicating that nucleolar targeting is intrinsic to P3 rather than dependent on GFP fusion) (Fig. 1b). Isoform-specific localization in infected cells cannot currently be resolved due to shared sequences between P-protein isoforms and their differential expression levels; therefore, localization was examined using GFP-tagged isoforms in transfected cells. Nish P3 accumulated strongly in nucleoli, similar to CVS, whereas P1 and P2 were excluded from the nucleus, and P4 and P5 also showed clear nucleolar accumulation. Quantitative image analysis of fluorescence intensity ratios between the nucleus and cytoplasm (Fn/c), calculated as previously described [16,18,20], supported these observations: P1 and P2 localized almost exclusively to the cytoplasm (Fn/c<0.2), P3 and P4 accumulated in the nucleus (Fn/c ≈ 1.5), and P5 showed intermediate distribution (Fn/c ≈ 0.6) (Fig. 1c).

To test whether Nish P1 and P2 could access nucleoli when retained in the nucleus by inhibition of nuclear export, we treated cells with leptomycin B (LMB), an exportin-1/CRM1 inhibitor of P-protein nuclear export [10,12]. As reported for CVS P1 [17], LMB caused Nish P1 to accumulate in the nucleus but remain excluded from nucleoli. Similarly, LMB drove P2 into the nucleus (Fn/c ≈ 3), yet it too was nucleolar-excluded (Fig. 1b, c). Quantitative analysis confirmed that P1 and P2 had nucleolar-to-nuclear ratios <0.8, comparable to GFP alone, whereas P3–P5 accumulated strongly in nucleoli (Fnu/n 1.4–1.6) (Fig. 1d). Thus, exclusion from nucleoli is a conserved property of P1 across strains and extends to P2, while truncation of the N-terminus confers a gain-of-function in nucleolar localization to P3–P5. These localization phenotypes also align with P-protein’s roles in microtubule (MT) association and bundling, processes that can be promoted by LLPS of MT-associated proteins [21]: P3–P5 bundle MTs, whereas P1 and P2 do not [18]. Together with our recent findings [17], these results support that the ability of P-protein isoforms to target diverse host membraneless bodies (nucleoli, nuclear bodies and MT bundles) is mechanistically linked.

The genus *Lyssavirus* currently comprises at least 17 recognized species, classified into two major phylogroups (I and II) based on genetic and serological divergence [22]. Phylogroup I includes the majority of species, such as RABV, silver-haired bat rabies virus (SHBRV), Australian bat lyssavirus (ABLV), Duvenhage virus (DUVV), and European bat lyssaviruses 1 and 2 (EBLV-1 and EBLV-2). Phylogroup II comprises Lagos bat virus (LBV), Mokola virus (MOKV) and Shimoni bat virus. With the exception of MOKV, whose reservoir remains unknown, lyssaviruses are primarily maintained in bats. RABV is unique among lyssaviruses in being stably maintained in terrestrial carnivores, as well as in bats [23].

To investigate whether nucleolar targeting is conserved across lyssaviruses, we analysed representative members of phylogroups I and II, which contain the majority of known species and include all classical RABVs. Alignment of selected P-proteins indicated that not all lyssaviruses encode P3, as several lack a start codon at the relevant position (e.g. ABLV, EBLV-1, DUVV) (Fig. S1, available in the online Supplementary Material). We, therefore, restricted analysis to P3 proteins containing the internal start codon. Sequence alignment revealed strong conservation of the P-protein C-terminal domain (CTD) across all analysed viruses, including complete conservation of residues K214 and R260 previously shown to be essential for nucleolar retention [16], indicating that all P3 proteins retain known nucleolar targeting determinants. GFP-fused P3s from phylogroup I (CVS, Nish, SHBRV, a bat-associated field strain of RABV and EBLV-2) and phylogroup II (LBV, MOKV) were expressed in HeLa cells and examined by CLSM. Subcellular localization varied between species: CVS, Nish, EBLV-2 and MOKV P3s were mainly nuclear, whereas SHBRV and LBV were more cytoplasmic (Fig. 2a). Despite this variation, nucleolar accumulation was consistently observed for phylogroup I members, whereas MOKV P3, though nuclear, was reduced or excluded.

**Fig. 2.**
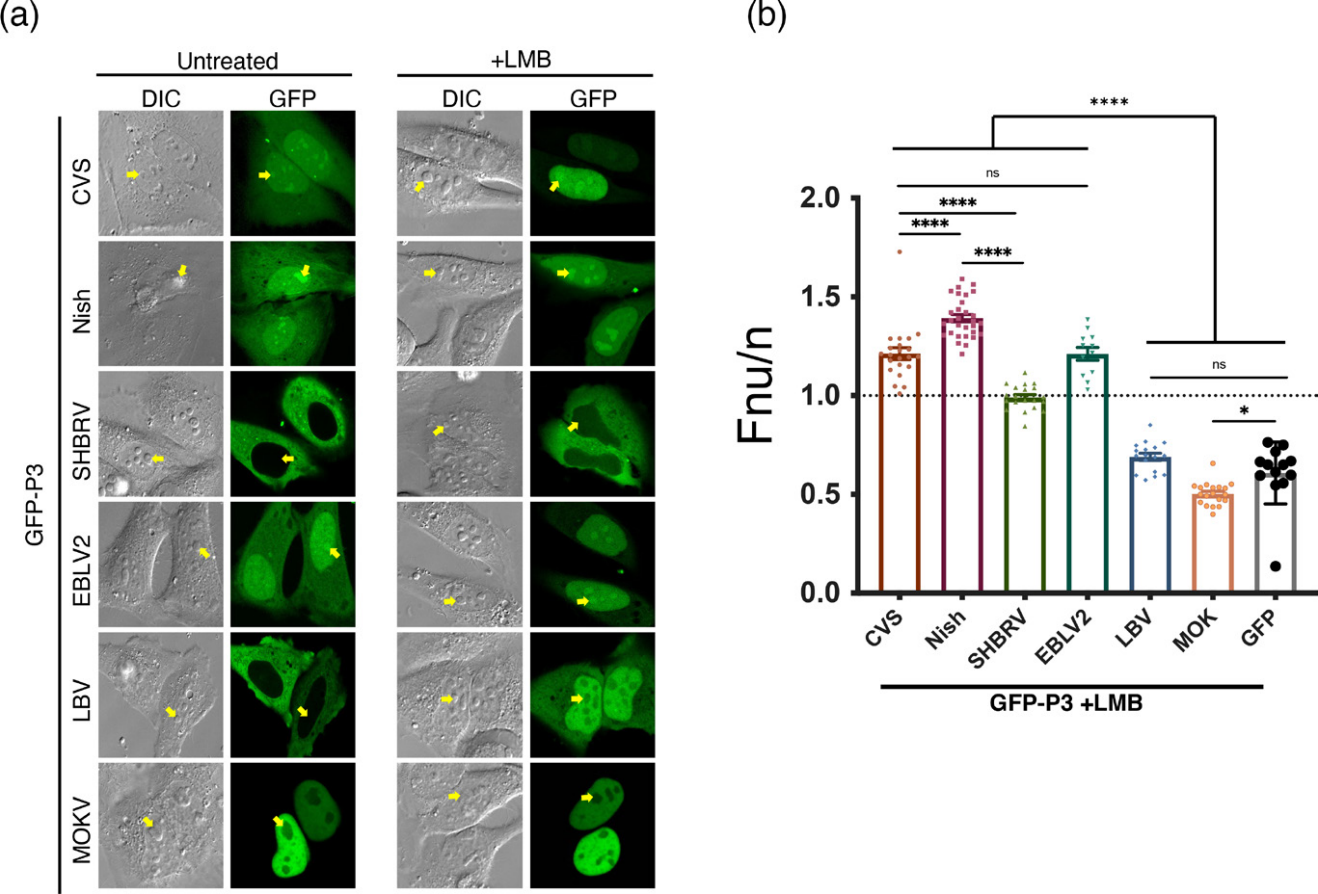
Lyssavirus P3 nucleolar targeting is conserved in phylogroup I but absent from phylogroup II. (**a**) HeLa cells were transfected with GFP-fused P3 proteins from the indicated lyssaviruses (CVS, Nish, SHBRV, EBLV-2, LBV, MOKV) and imaged live 24 h post-transfection by CLSM as in Fig. 1. Cells were treated with or without 5 µM LMB for 4 h. Panels show DIC (left) and GFP fluorescence (right). Yellow arrows indicate nucleoli. (**b**) Quantitative analysis of LMB-treated cells such as those in a. Fnu/n were determined in ImageJ from ≥12 cells per condition. Data represent mean±sem from one representative assay and are consistent with three independent experiments. Statistical significance was assessed using Student’s t-test; *P<0.05, **P<0.01, ***P<0.001, ****P<0.0001.

To account for differences in nucleocytoplasmic trafficking, we treated cells with LMB to force proteins into the nucleus. LBV P3 accumulated in the nucleus but remained excluded from nucleoli, similar to MOKV (Fnu/n<0.6) (Fig. 2b). SHBRV P3 also showed increased nuclear levels after LMB but still targeted nucleoli less efficiently than other phylogroup I members (Fnu/n ≈ 1 vs >1.2). Thus, while nuclear localization varies across species, nucleolar targeting is conserved among phylogroup I P3 proteins but absent from phylogroup II. Consistent with CVS and Nish strains, P1 proteins from multiple lyssaviruses, including ABLV, DUVV, MOKV and SHBRV, remained excluded from nucleoli even when forced into the nucleus with LMB (Fig. S2). These findings suggest that P1 exclusion from host membraneless compartments is broadly conserved, in contrast to P3, and may reflect P1’s essential role in replication within LLPS-driven viral factories (Negri bodies) [24].

We next asked whether interactions with nucleolar proteins are similarly conserved. CVS P3 has previously been shown to bind NCL [14] and Treacle [16], and more recently, NPM1 and NOLC1 were identified as additional interactors [17]. Co-immunoprecipitation (co-IP) assays of GFP-tagged P3 proteins expressed in HEK-293T cells, performed as previously described [17,25], revealed that interactions with NCL and NPM1 were broadly conserved across phylogroup I and II members, although reduced in the latter, with LBV showing especially weak binding (Fig. 3). In contrast, the Treacle association was restricted to RABV strains: CVS and Nish bound efficiently, and SHBRV bound most strongly. NOLC1 binding was observed for phylogroup I P3 proteins but was significantly reduced or absent in phylogroup II.

**Fig. 3.**
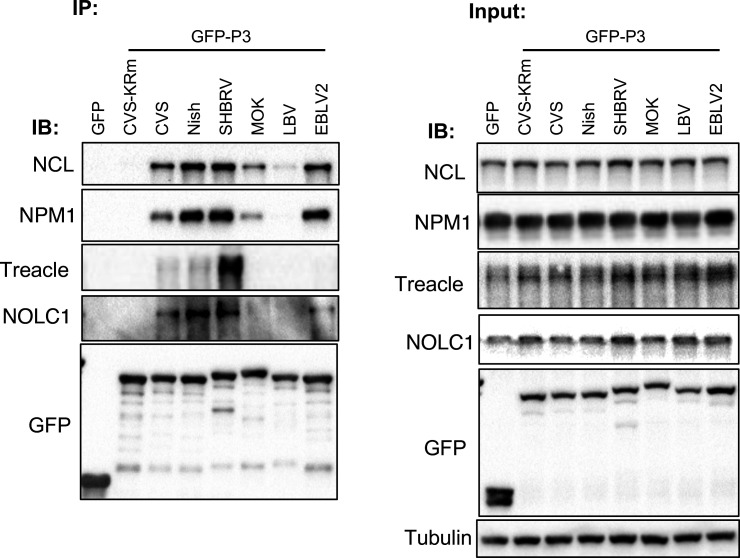
RABV P3 binds multiple nucleolar proteins, with variable conservation among lyssaviruses. GFP-tagged P3 proteins from the indicated lyssaviruses were expressed in HEK-293T cells and analysed by co-IP with GFP-trap as previously described [17,25], followed by immunoblotting (IB) with antibodies against NCL (CST, Cat# 14574; IB 1:1000), NPM1 (ThermoFisher, Cat# 32-5200; IB 1:2000), Treacle (ProteinTech, Cat# 11003-1-AP; IB 1:2000) or NOLC1 (ProteinTech, Cat# 11815-1-AP; IB 1:2000). GFP alone and the nucleolar-impaired mutant GFP–P3-KRm (K214A/R260A) were included as negative controls, as neither localizes to nucleoli. IP samples are shown in the left panels and corresponding input samples in the right panels. IB, immunoblotting.

These findings raise the question of why nucleolar targeting is maintained in phylogroup I but not phylogroup II lyssaviruses. P3 inhibition of rRNA biogenesis and the requirement for NCL and Treacle in virus production are established [14,16], but the functional significance of interactions with other nucleolar proteins such as NPM1 and NOLC1 remains unclear. NCL, Treacle, NPM1 and NOLC1 are all central to ribosome biogenesis and are common viral targets implicated in controlling rRNA transcription, nucleolar stress responses and host gene expression [16,26,29]. In contrast, divergence in Treacle and NOLC1 association may underlie lineage-specific adaptations. Perhaps, phylogroup II lyssaviruses have evolved alternative strategies or do not rely on nucleolar functions to the same extent, given their distinct host or reservoir dynamics. We note that our analysis focuses on the P-protein and its isoforms and, therefore, does not formally exclude the possibility that phylogroup II lyssaviruses engage nucleolar pathways via other viral proteins, although no such mechanisms have yet been described.

At the strain level, P-protein isoform expression may also vary among RABV strains. For example, sequence analysis indicates that SHBRV lacks a methionine corresponding to the P4 initiation codon, suggesting that P4 expression may be reduced or absent in this naturally circulating strain. More broadly, isoform-specific P-protein expression across wild RABV isolates remains poorly characterized, and quantitative variation in isoform output and subcellular targeting may contribute to strain-specific tuning of host interactions.

The observation that P3, but not P1, engages host LLPS compartments, including nucleoli, nuclear bodies and MT bundles, further suggests that these interactions are mechanistically linked, consistent with our recent work showing that RNA binding regulates P3 condensate targeting, whereas P1, despite LLPS propensity, lacks RNA-binding capacity [17]. P1 appears excluded from host condensates but retained in Negri bodies [17,24], perhaps reflecting a division of labour whereby P1 is dedicated to replication and P3 to host subversion. Together, this highlights nucleolar targeting as a condensate-based interface for conserved and divergent virus–host interactions, shaping lyssavirus multifunctionality and evolution, and points to nucleolar interactions as a potential vulnerability for therapeutic intervention.

## Supplementary material

10.1099/jgv.0.002214 Uncited Supplementary Material 1.

## Abbreviations

ABLV: Australian bat lyssavirus
CLSM: confocal laser scanning microscopy
co-IP: co-immunoprecipitation
CTD: C-terminal domain
DUVV: Duvenhage virus
EBLV: European bat lyssaviruses
LBV: Lagos bat virus
LLPS: liquid–liquid phase separation
LMB: leptomycin B
MOKV: Mokola virus
MT: microtubule
NCL: nucleolin
NES: nuclear export sequence
Nish: Nishigahara
NLS: nuclear localization signal
NOLC1: nucleolar and coiled-body phosphoprotein 1
NPM1: nucleophosmin
P-protein: phosphoprotein
RABV: rabies virus
SHBRV: silver-haired bat rabies virus
