# Supplementary material for: Nucleolar targeting of lyssavirus P-protein is isoform- and phylogroup-specific
Source: J Gen Virol. 2026 Jan 16;107(1):002214. doi: 10.1099/jgv.0.002214 (PMC12811153; doi:10.1099/jgv.0.002214)
Supplement: Uncited Supplementary Material 1. [file jgv-107-02214-s001.pdf]

Supplementary Data for:

Nucleolar targeting of lyssavirus P-protein is isoform- and phylogroup-specific

Gregory W. Moseley[1*#], Yilin Zhang[2], Cassandra T. David[1], Stephen M. Rawlinson[1*#]

[1] Department of Microbiology, Biomedicine Discovery Institute, Monash University, Clayton, VIC, 3800, Australia

[2] Department of Biochemistry and Molecular Biology, Bio21 Molecular Science and Biotechnology Institute, The University of Melbourne, Melbourne, 3052, Australia

Supplementary Figure 1

```
                    P1                P2                               P3
CVS      MSKIFVNPSAIRAGLADLEMAEETVDLINRNIEDNQAHLQGEPIEVDNLPEDMKRLHLDD   60
Nish     MSKIFVNPSAIRAGLADLEMAEETVDLINRNIEDNQAHLQGEPIEVDSLPEDMSRLHLDD   60
SHBRV    MSKIFVNPSAIRAGLADLEMAEETVDLIARNIEDNQAHLQGEPIEVDSLPEDMRRLQLDD   60
ABLV     MSKIFVNPSAIRAGMADLEMAEETVDLINRNIEDNQAHLQGEPIEVDSLPEEIGKLNINE   60
EBLV2    MSKIFVNPSAIRAGLADLEMAEETVDLVNKNIEDNQAHLQGEPIEVDALPEDMSKLQISE   60
EBLV1    MSKIFVNPSALRSGLADLEMAEETVDLVNKNMEDSQAHLQGIPIDVETLPEDIKRLRIAD   60
DUVV     MSKIFINPSDIRSGLADLEMAEETVDLVNRNMEDSQAHLQGVPIDVETLPEDIRRLHITD   60
LBV      MSKDLVHPSLVRAGYVELEMADETADVVNLTIASNQAHLQGEPFYVDSLPEDMNRMRLAE   60
MOKV     MSKDLVHPSLIRAGIVELEMAEETTDLINRTIESNQAHLQGEPLYVDSLPEDMSRLRIED   60
         *** :::** :*:* .:****:**.*::  .: ..****** *: *: ***:: ::.: :

                     P4          P5
CVS      EKSSNLGEMVRVGEGKYREDFQMDEGEDPNLLFQSYLDNVGVQIVRQMRSGERFLKIWSQ   120
Nish     GKLPDLGRMSKAGEGRHQEDFQMDEGEDPSLLFQSYLDNVGVQIVRQMRSGERFLKIWSQ   120
SHBRV    TKPSGPGEIAATGESKCQEDFQMDEAEDPALLFQSYLDNVGIQIVRQMKSGERFLKIWSQ   120
ABLV     AKPQSFENNPIDIDGRMNEDFQMKEVEDPSIQFQSYLDNIGIQIVRKMKTGERFFKIWSQ   120
EBLV2    RRPAQFTDNTGGKEEGSDEDFYMAESEDPYIPLQSYLEGVGIQLVRQMKTGERFFKIWSQ   120
EBLV1    YKQGQQEEDASRQEEGEDEDFYMTESENSYVPLQSYLDAVGVQIVRKMKTGDGFFKIWAQ   120
DUVV     PQASLRQEMIEEQKHQEDEDFYLTERENPLSSFQTHLDAVGLRIVRKMKTGEGFFKIWSQ   120
LBV      TSGKMEVAEDDEDDCSSEDEFYLPKGQDPLVPFQGFLEEVGARIVKRMKSGEGFFKIWST   120
MOKV     KSRRTKTEEEERDEGSSEEDNYLSEGQDPLIPFQNFLDEIGARAVKRLKTGEGFFRVWSA   120
                      .    ::  : : ::    :* .*: :* : *::::::*: *:::*:

CVS      TVEEIVSYVTVNFPNPPRRSSEDKSTQTTGRELKKET-TSAFSQRESQPS--KARMVAQV   177
Nish     TVEEIISYVTVNFPNPSGRSSEDKSTQTTSQEPKKET-TSTPSQRKSQSL--KSRTMAQT   177
SHBRV    TVEEIISYVTVNFPSLPGKSTEDKATQTASRELKKET-VSAPSQRDSQSS--KTKVAAQT   177
ABLV     TVEEIISYVGANFPNPSGKTTESKSTQTTPKKVKPEP-PSAPTEKPEQLS--RTSMAPET   177
EBLV2    AVEEIISYVTVHFPMPLGKSTEDKSTQTPEEKFKPSP-QQAVTKKESQSS--KIKTISQE   177
EBLV1    AVEDIVSYVATNFPAPVNKPHADKSTQTTLEKVKQATSSSAPSKREGPSS--NVNLDSQE   178
DUVV     AVEDIVSYVALNFSIPVNKLFEDKSTQTVTEKSQQVSASSAPSRHDKSSQ--GARVDSKD   178
LBV      MTEDIKGYVLTNVMKTSSKTVENRQTQTDKQLSSNLPQELTPPKREEALN-PKKAEERPK   179
MOKV     LSDDIKGYVSTNIMTSGERDTKSIQIQTEPTASVSSGNESRHD-SESMHDPNDKKDHTPD   179
           ::* .**  :.     :   .   **

                                               K214
CVS      APGPPALEWSATNEEDDLSVEAEIAHQIAESFSKKYKFPSRSSGIFLYNFEQLKMNLDDI   237
Nish     ASGPPSLEWSATNEEDDLSVEAEIAHQIAESFSKKYKFPSRSSGIFLYNFEQLKMNLDDI   237
SHBRV    ASGPPALEWSTANEEDDPSVEAEIAHQIAESFSKKYKFPSRSSGIFLYNFEQLKMNLDDI   237
ABLV     TSGPLALDWSATNDDDDVSVEAEIAHQIAESFSKKYKFPSRSSGIFLYNFEQLKMNLDDI   237
EBLV2    SSGPPALEWSTTNDEENASVEAEIAHQIAESFSKKYKFPSRSSGIFLFNFEQLKMNLDDI   237
EBLV1    SSGPPGLDWAASNDEDDGSIEAEIAHQIAESFSKKYKFPSRSSGIFLWNFEQLKMNLDDI   238
DUVV     ASGPPALDWTVSNEADDESVEAEIAHQIAESFSKKYKFPSRSSGIFLWNFEQLKMNLDEI   238
LBV      PSKAPEVSWTNETE-EVNDIEGEVAHQVAESFSKKYKFPSRSSGIFLWNFEQLKMNLDDI   238
MOKV     HDVVPDIESSTDKG-EIRDIEGEVAHQVAESFSKKYKFPSRSSGIFLWNFEQLKMNLDDI   238
               :. :  .  :  .:*.*:***:*******************:**********:*

                           R260
CVS      VKEAKNVPGVTRLAHDGSKIPLRCVLGWVALANSKKFQLLVEADKLSKIMQDDLNRYTSC   297
Nish     VKEAKNVPGVTRLAHDGSKLPLRCVLGWVALANSKKFQLLVEANKLNKIMQDDLNRYASC   297
SHBRV    VKESKNVPSVTRLAHDGSKLPLRCVLGWVALAKSKKFQLLVEPDKLNKIMQDDLNRYAFP   297
ABLV     VKEARGVPGIIRLAKEGLRLPLRCILGWVASSHSKKFQLLVGSEKLSKIMQDDLNRYMSC   297
EBLV2    VKEAKKIPGVVRLAQDGFRLPLRCILGGVGSVNSKKFQLLVNSDKLGKIMQDDLNRYLAY   297
EBLV1    VREVKGIPGVTRMARDGMKLPLRCMLGSVASNHSKRFQILVNSAKLGKLMQDDLNRYLAY   298
DUVV     VREVKEIPGVIKMAKDGMKLPLRCMLGGVASTHSRRFQILVNPEKLGKVMQEDLDKYLTY   298
LBV      VKASMNIPGVEKIAEKGGKLPLRCILGFVALDSSKRFRLLADTDKVARLMQDDINSYMAR   298
MOKV     VKAAMNVPGVERIAEKGGKLPLRCILGFVALDSSKRFRLLADNDKVARLIQEDINSYMAR   298
         *:    :*.: ::*..* ::****:** *.   *::*::*.   *: :::*:*:: *

CVS      -------    297
Nish     -------    297
SHBRV    -------    297
ABLV     -------    297
EBLV2    -------    297
EBLV1    -------    298
DUVV     -------    298
LBV      LEDVDRN    305
MOKV     LEEAE--    303
```

(In each block, CVS, Nish and SHBRV are bracketed as "Rabies strains"; CVS to DUVV as "Phylogroup I"; LBV and MOKV as "Phylogroup II".)

<u>Supplementary Figure 1.</u> Sequence alignment of lyssavirus P-proteins used in this study. Amino acid sequences of P-proteins from representative lyssaviruses were aligned using Clustal Omega. Strains analysed were CVS-11 (GQ918139.1), Nishigahara (AB044824.1), SHBRV (AY705373.1), EBLV-2 (NC_009528.2), Mokola virus (MOKV; NC_006429.1), Lagos bat virus (LBV; NC_020807.1), Duvenhage virus (DUVV; JN986749.1), Australian bat lyssavirus (ABLV; NC_003243.1), and EBLV-1 (KP241939.1). Start codons (methionines, Met) corresponding to isoforms P1–P5 are indicated in yellow. Additional Met residues within the N-terminal 1–83 amino acid region that may potentially give rise to alternative truncated isoforms are highlighted in green. Conserved residues K214 and R260, mutation of which to alanine (KRm) abolishes nucleolar targeting, are highlighted in cyan. Strains belonging to RABV are marked on the right in red, with their corresponding phylogroup (I or II) indicated. Clustal Omega annotation symbols are shown below the alignment: "*" indicates fully conserved residues, ":" indicates strong conservation, and "." indicates weaker conservation. Numbers on the right denote amino acid positions.

Supplementary Figure 2

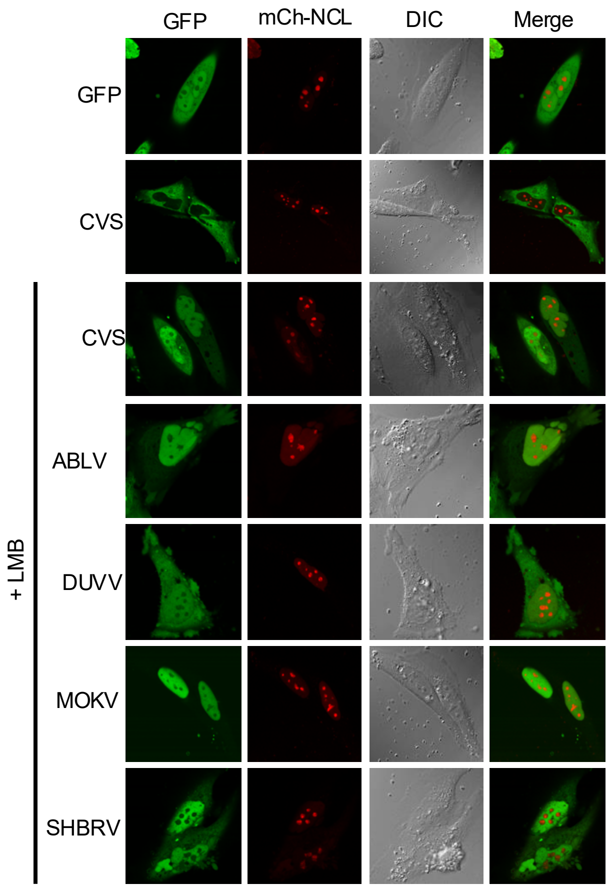

<u>Supplementary Figure 2</u>. P1 proteins from multiple lyssaviruses remain excluded from nucleoli.
HeLa cells were co-transfected with GFP-tagged P1 proteins from the indicated lyssaviruses (CVS, Nish, ABLV, DUVV, MOKV, SHBRV) and mCherry-nucleolin (mCh-NCL) as a nucleolar marker. Live cells were imaged 24 h post-transfection by CLSM (Leica SP5, 60× oil objective). Panels show GFP (left), mCh-NCL (second column), DIC (third column), and merged GFP/mCh-NCL channels (right). P1 proteins accumulated in the cytoplasm or nucleus but were consistently excluded from nucleoli, in contrast to nucleolar accumulation of P3 isoforms (see Figures 1 and 2). Representative images from ≥ 13 fields of view.
